# A perturbation approach for ultrafast calculation of RF field enhancements near medical implants in MRI

**DOI:** 10.1038/s41598-022-08004-7

**Published:** 2022-03-10

**Authors:** Peter R. S. Stijnman, Bart R. Steensma, Cornelis A. T. van den Berg, Alexander J. E. Raaijmakers

**Affiliations:** 1 Computational Imaging Group for MRI diagnostics and therapy, Centre for Image Sciences UMC Utrecht, Utrecht, Utrecht, 3584 CX The Netherlands; 2 Department of Biomedical Engineering, Eindhoven University of Technology, Eindhoven, Brabant 5612 AZ The Netherlands

**Keywords:** Applied physics, Imaging techniques, Computational science

## Abstract

Patients with medical implants often are deprived of magnetic resonance imaging examination because of safety risks. One specific risk is the enhancement of the radiofrequency fields around the medical implant potentially resulting in significant tissue heating and damage. The assessment of this enhancement is a computationally demanding task, with simulations taking hours or days to converge. Conventionally the source of the radiofrequency fields, patient anatomy, and the medical implant are simulated concurrently. To alleviate the computational burden, we reformulate a fast simulation method that views the medical implant as a small perturbation of the simulation domain without the medical implant and calculates the radiofrequency fields associated with this perturbation. Previously, this method required an extensive offline stage where the result is intractable for large simulation domains. Currently, this offline stage is no longer required and the method is completely online. The proposed method results in comparable radiofrequency fields but is orders of magnitude faster compared to standard simulation technique; the finite-difference time-domain, the finite-sums, and the finite element methods. This acceleration could enable patient-specific and potentially online radiofrequency safety assessment.

## Introduction

**Figure 1:**
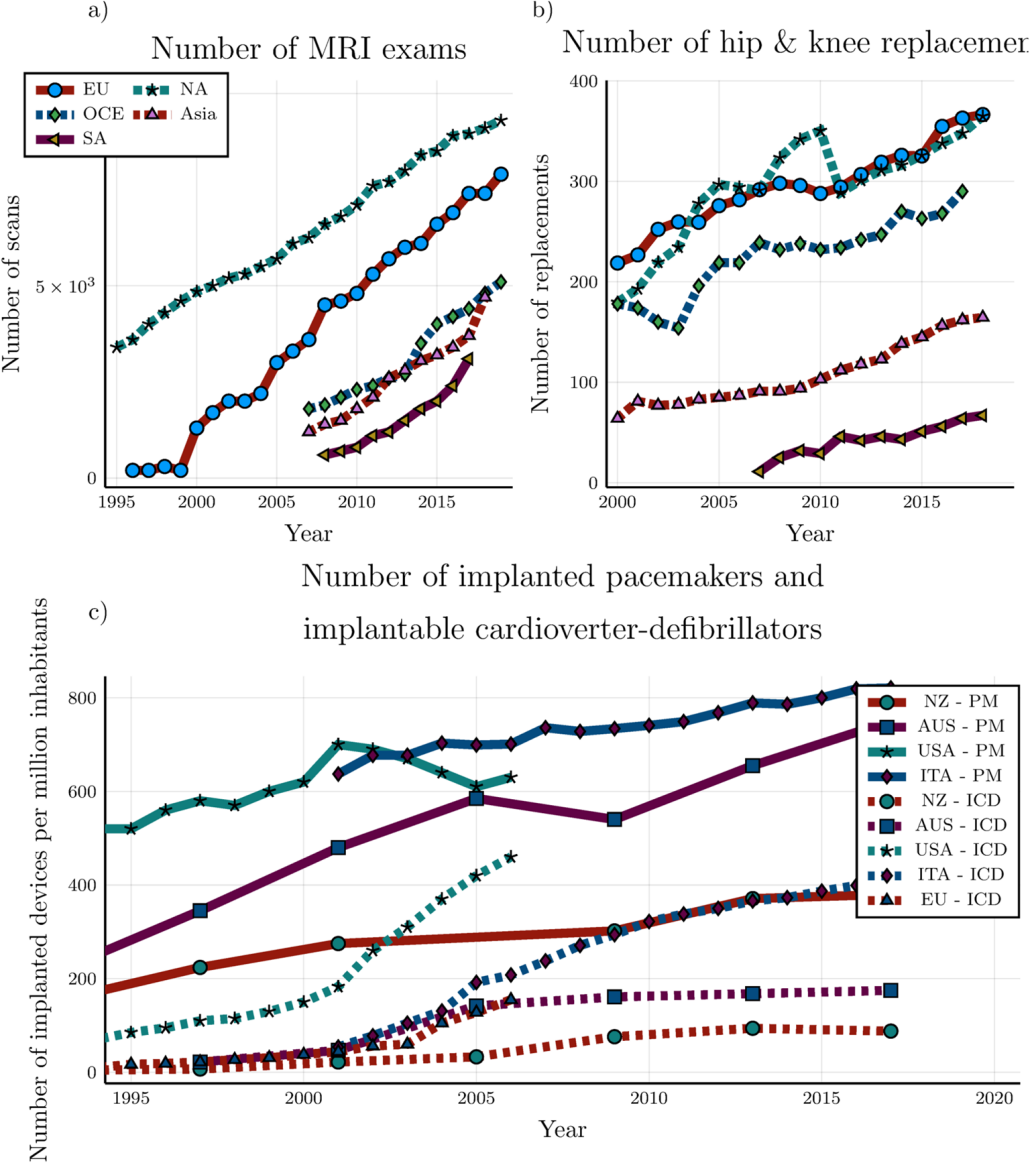
(**a**) The number of MRI scans performed per 100.000 inhabitants. (**b**) The number of hip and knee replacements per 100.000 inhabitants. Data presented for the 21 indicated countries (OECD (2021), Magnetic resonance imaging (MRI) exams (indicator). https://data.oecd.org/healthcare/magnetic-resonance-imaging-mri-exams.htm (Accessed on 01 August 2021)). (**c**) The number of implanted pacemakers (PM) and implantable cardioverter-defibrillators (ICD) per 1 million inhabitants in the indicated countries^2–5^.

Magnetic resonance imaging (MRI) is one of the main medical imaging modalities and has become indispensable for diagnosis, treatment planning, and patient follow-up studies. The number of people requiring an MRI examination is steadily increasing every year as can be seen in Fig. 1^1^. This is in part because of the excellent soft-tissue contrast provided by this imaging modality. Based on the principle of nuclear magnetic resonance, MRI requires three types of magnetic fields: a permanent strong magnetic field (1.5 or 3 T), rapidly switching magnetic gradient fields for spatially encoding the signal, and radiofrequency (RF) fields generated by an RF coil or antenna to excite the atomic nuclei to create the signal. This multitude of magnetic fields poses a severe safety risk for patients with medical implants. Also, this patient category is rapidly growing. For example, from Fig. 1(b) and (c) it can be observed that the number of hip and knee replacement, and the number of pacemakers (PMs) and implantable cardioverter-defibrillators (ICDs) surgeries is increasing annually^2–5^. Both MRI investigations and medical implants are more prevalent in the aging population; it is estimated that 50–75% of patients with PMs and ICDs will require an MRI within the lifetime of the device^6^.

Patients with medical implants can be excluded for an MRI examination because of the many safety concerns that exist when introducing metal inside the MRI environment^7–9^. The safety concerns include displacement, malfunctioning, and heating of the medical implant all of which can harm the patient under examination^10–12^. This has occurred in a handful of instances with detrimental outcomes^13–17^. Furthermore, in the USA alone around 300 cases of adverse events involving MRI and medical implants are reported to the FDA annually^18^. When these patients are scanned, extra precautions have to be taken; an individual risk-benefit analysis, extra monitoring and experienced physicians are mandatory, and an informed consent by the patient is required. Since a lot of smaller hospitals either do not have the equipment or the experience these patients are often referred to another hospital or not scanned at all.

To avoid depriving patients of the diagnostic capabilities of MRI, implant manufacturers have been developing increasingly more products that are MRI-compatible. To facilitate this, implant manufacturers, MRI vendors, and the scientific community have set up a technical specification for passive implanted medical devices like surgical plates or vascular stents; ASTM F2182^19^, and for active implanted medical devices like pacemakers or neurostimulators; ISO/TS 10974^20^. Depending on the results of the prescribed tests and simulations included in this technical specification, an implant can obtain a label stating it is MRI safe, MRI conditional, or MRI unsafe. When a medical implant is labeled, MRI conditional limitations are placed on the MRI sequence parameters (e.g. maximum RF power). These limitations decrease the image quality and/or increase the required scan time.

One particularly demanding and challenging safety aspect of this technical specification is the RF safety testing. The RF fields that are generated during an MRI examination can induce currents in metallic implants which are excellent conductors. The induced currents cause charge accumulation at the interfaces between the implant and the tissues surrounding the implant. The charge accumulation creates conservative electric fields in those tissues and thereby enhances the RF electric field. This enhancement creates locations where the temperature is drastically increased, potentially causing tissue burns inside the patient or even excessive brain damage^17^.

The potentially dangerous enhancement of the electric field can be calculated using for example finite difference time domain (FDTD) or finite element method (FEM) simulations. The electric field inside the patient results in power deposition because of the conductivity of the tissue. The amount of dissipated power is expressed by the local specific absorption rate (SAR), which is the power deposition divided by the mass density of the tissue.

To correctly assess the RF safety of a medical implant a wide variety of patient anatomies^21^ and RF coils^22–27^ should be simulated. Furthermore, the location of the medical implant inside the patient anatomy and the patient position within the RF coil are important factors for the SAR increase. A single simulation can take up to hours or even days to converge even with GPU acceleration^28,29^. Therefore, the RF safety assessment of implants is typically done using simplified scenarios because the full analysis of every possible combination of patient anatomy, implant location inside the patient, patient position inside the scanner, and RF coil (so-called Tier 4 approach in ISO/TS 10974^20^) is computationally too demanding.

These simplified scenarios are overly conservative to ensure safety and therefore often result in medical implants being conditionally safe. However, the resulting scanning constraints are for the largest patient group overly conservative because these constraints are determined for the worst-case scenario. This entails the worst implant position inside the patient in the worst position in the scanner while having the worst-case patient anatomy. Therefore, if the patient-specific RF interaction of the implant could be characterized before the patient arrives for their MRI examination, these conservative scanning constraints could be relaxed for the vast majority of patients. Furthermore, when the characterization of the RF interaction can be done within the time span of a few minutes the RF safety assessment could be performed online at the scanner. In addition, subject-specific RF safety assessment could make the large group of patients that carry non-labeled or multiple implants eligible again for MRI examination. Currently, the simulation process for RF safety assessment is too slow to be performed before a patient is scheduled for an MRI examination.

In previous work, a method has been described to accelerate the simulation process significantly^30,31^. In this method, if the RF field distribution without the implant is known, the introduction of the implant into the setup can be considered a small perturbation of the system. We consider a perturbation small if it does not perturb the source of the RF fields (i.e. the RF coil). The RF field distributions arising from this small perturbation can be calculated on the small domain where the implant is present in comparison to the entire simulation domain including the RF coil and patient anatomy. This method requires an extensive offline precomputation stage dependent on the frequency, patient anatomy, and location of the implant. Afterward, the method can calculate the RF fields within the small domain where the problematic heating will occur in mere seconds compared to hours using standard simulation techniques.

The proposed method bears some resemblance to the Tier 3 approach in the ISO/TS 10974^20^ which uses the transfer function, together with the RF field distribution without the implant present. The transfer function is a simplified model of the electromagnetic interaction of the implant with the electric RF field distribution along the implant. Furthermore, the transfer function as in the ISO/TS 10974 is only defined for elongated implant such as pacemaker leads. For three-dimensional implants using the Tier 3 approach is not feasible, whereas the proposed method is.

Unfortunately, the proposed method cannot be used practically. The result of the offline precomputation stage requires such an excessive amount of memory storage that the maximum calculable size of the implant is rather small. For example, when a human head model, with average dimensions of 15.2 cm by 18.6 cm by 11.2 cm (i.e. from top of the head down to the eyes), is discretized on a 1 mm isotropic resolution this would require around 650 TB of memory to store all the simulated field distributions in single precision complex float numbers. Furthermore, using an FDTD solver running on a single GPU would take almost 3 years to complete, at 10 secs per simulation^31^.

In this work, a rigorous adaptation of the earlier method is presented by which the drawbacks of the previous method are overcome^30,31^. The result is an ultrafast calculation method of RF field enhancement by medical implants in MRI. The presented method is fast enough to be used for patient specific and/or online RF safety assessment. This would improve image quality and/or reduce scan time for most implants with a conditional MRI safety label. Apart from the patient specific and online possibilities for RF safety assessment, the presented method also enables RF field calculations for the highest standard of RF safety assessment (Tier 4 in ISO/TS 10974) in a significantly accelerated fashion.

Although the method has been developed for MRI implant safety assessment, it is suitable for any application area where electromagnetic simulations (EM) are being used. If a calculated EM field distribution is perturbed by a small change in the simulation domain, the presented method allows for an unprecedented calculation speed of the new field distribution, without the need for an extensive offline precomputation stage.

## Results

**Figure 2:**
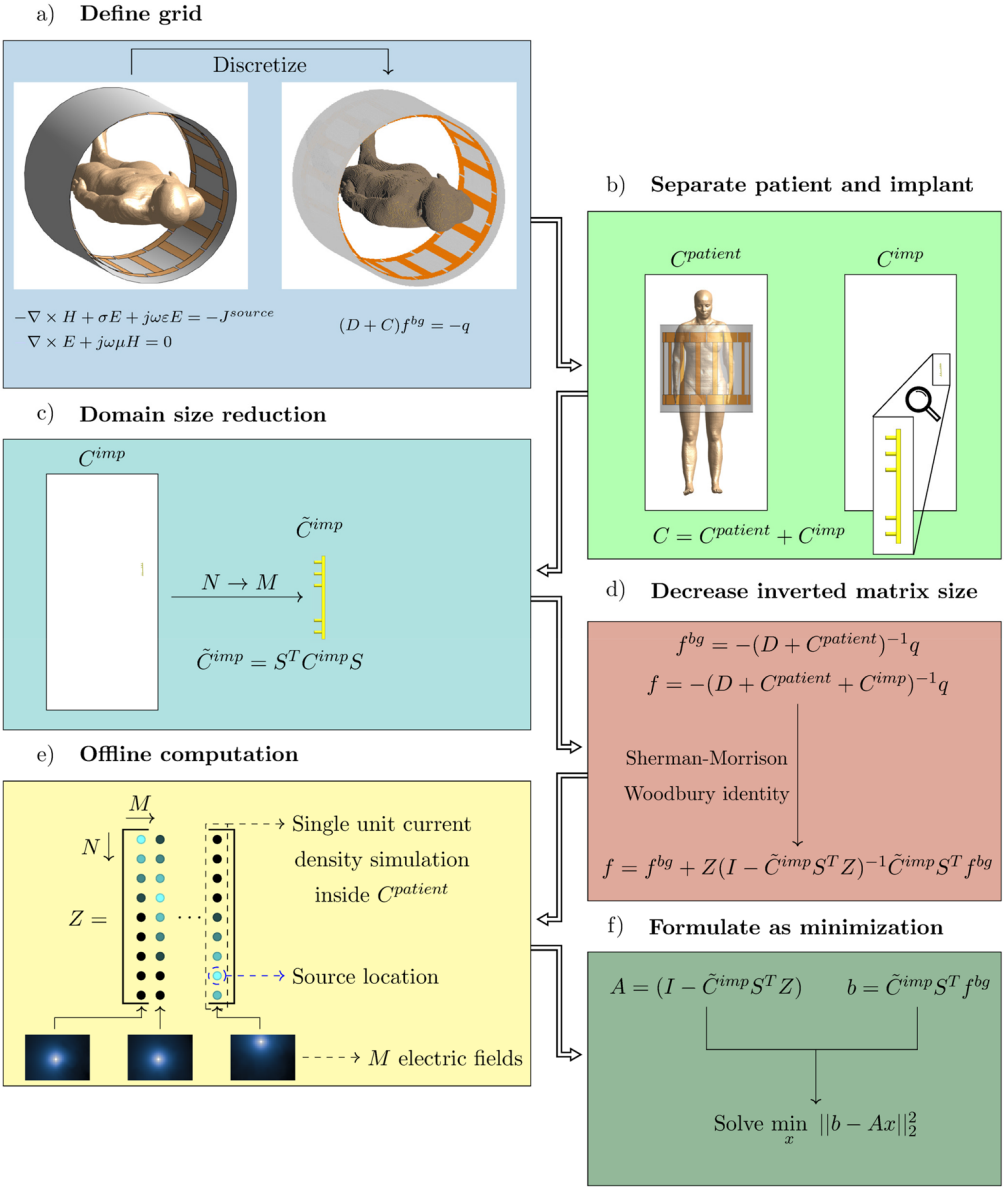
Schematic visualization of the prior method still using the library matrix. (**a**) The RF coil, patient anatomy, and the implant are discretized on a cubic grid. (**b**) The dielectric of patient anatomy and the implant are separated. (**c**) The implant is only present on a small domain in the simulation, therefore we can decrease the domain size of the computation to only those locations where the implant is present. (**d**) using the Sherman–Morrison–Woodbury identity we can do a low rank update to the RF field distribution without the implant. As a result of the domain reduction, the matrix inverse can now be calculated. (**e**) before we can execute this calculation the so-called library matrix containing the electric field response of unit current densities at all possible implant locations is required. (**f**) once this is available we can compute the field distribution online by casting the matrix inversion as a minimization problem.

First, the proposed calculation method is presented. In Fig. 2 a visual overview of the prior methodology is shown. For more detail, the derivation of this method is described in the “Methods” section. Subsequently, the RF field distribution for four common medical implants is calculated using the proposed method and compared to a full FDTD simulation.

### Theory to circumvent the offline calculation

In this work, we will start from the equation given in^30,31^ that describes a perturbation of the RF fields as a result of a medical implant, given by

1 $$\begin{aligned} f = f^{bg} +Z(I - {\tilde{C}}^{imp}S^TZ)^{-1}{\tilde{C}}^{imp}S^Tf^{bg}, \end{aligned}$$

here *f* and $f^{bg}$ contain the electric and magnetic RF fields concatenated in a vector of length *N*, where *N* is the number of edges inside the simulation domain, when the implant is and is not present respectively. *I* is the identity matrix of size *M* by *M*, where *M* is the number of edges the implant occupies inside the discretized simulation domain (i.e. the size of the implant). The electric properties and the location of the implant are defined by ${\tilde{C}}^{imp}$ (*M* by *M*) and $S^T$ (*M* by *N*) respectively. The last matrix, *Z*, is the so-called library matrix that by definition contains the RF field distributions of a source, $J = 1 A/m^2$, within the patient anatomy. Each column, *z*, inside the library matrix corresponds to a source located at a single edge indicated by the column of the support matrix *S* according to

2 $$\begin{aligned} Z = [z_1,\ldots ,z_M], \end{aligned}$$

where the columns are *N* elements long. The original method prescribes computing this library matrix beforehand by use of numerical simulations which results in unmanageable memory requirements and precomputation times, as mentioned in the introduction. In addition, the resulting matrix *Z* is patient anatomy-, discretization-, and frequency-specific.

To tackle the issues of memory constraints and precomputation times, we propose an alternative procedure that circumvents the requirement of the library matrix entirely. This is achieved by solving the matrix inversion in Eq. (1) using a matrix-free approach. To explain the method, we take a closer look at the actual calculation procedure. To efficiently solve the matrix inversion in Eq. (1) the minimization problem

3 $$\begin{aligned} \underset{x}{\text {min}}\,\,\, ||b-Ax||_2^2, \end{aligned}$$

where $A = I - {\tilde{C}}^{imp}S^TZ$ and $b = {\tilde{C}}^{imp}S^Tf^{bg}$ is solved. The variable *x* for which we minimize represents the scattered current density as a result of the introduced implant. Equation (3) is iteratively minimized, therefore, during the minimization procedure the matrix *A* is not explicitly required, and only the matrix-vector product *A* with an estimate of *x* is required. Not having *A* explicit would reduce the memory constraints by approximately a factor *M*. Thus, if there is a function $g(x) = Ax$ that does not require *A* to be explicit, then Eq. (3) can also be reformulated as

4 $$\begin{aligned} \underset{x}{\text {min}}\,\,\, ||b-g(x)||_2^2. \end{aligned}$$

To find this function we start with working out the matrix-vector product according to

5 $$\begin{aligned} \begin{aligned} g(x)&= Ax,\\&= (I - {\tilde{C}}^{imp}S^TZ)x,\\&= x - {\tilde{C}}^{imp}S^TZx. \end{aligned} \end{aligned}$$

Here ${\tilde{C}}^{imp}$, $S^T$, and *x* are known and only the matrix-vector product of *Zx* is not readily available. To obtain the result of *Zx* we first substitute Eq. (2) into this matrix-vector product

6 $$\begin{aligned} \begin{aligned} Zx&= z_1x_1 + \cdots + z_Mx_M\\&= \sum _{j=1}^{M} z_jx_j. \end{aligned} \end{aligned}$$

Here it can be observed that *Zx* is the superposition of the unit current density field distributions in *Z* scaled with the present estimate of the current density distribution defined by *x*. These field distributions can efficiently be calculated using the volume integral equation method (VIE). The generated electric field ($E^t$) as a result of a source located within the patient anatomy can be expressed using the VIE method as an incident electric field ($E^{inc}$) generated by the source in a vacuum and a scattered electric field ($E^{sc}$) as a result of the patient anatomy following

7a $$\begin{aligned} E^t(\rho )= & {} E^{inc}(\rho ) + E^{sc}(\rho ), \end{aligned}$$

7b $$\begin{aligned} E^{inc}(\rho )= & {} -i\omega \mu _0\left( I + \frac{1}{k_b^2}\nabla \nabla \cdot \right) \int_{\rho^\prime  \in bg} G(\rho -\rho^\prime ) J(\rho^\prime )dV, \end{aligned}$$

7c $$\begin{aligned} E^{sc}(\rho )= & {} \left( I + \frac{1}{k_b^2}\nabla \nabla \cdot \right) \int_{\rho^\prime  \in bg} G(\rho -\rho^\prime ) C^{bg}(\rho^\prime ) E^t(\rho^\prime )dV. \end{aligned}$$

Note that we follow antenna theory here and in literature on the RF safety of implants the terminology for the electric fields is different^32,33^. The magnetic permeability and wavenumber in vacuum are denoted by $\mu _0$ and $k_b$, $\rho$ is the position vector, $C^{bg}$ contains the dielectric properties of the patient anatomy, *dV* is the volume of a single voxel, and the free-space Green’s function (*G*) is given by

8 $$\begin{aligned} G(\rho -\rho^\prime ) = \frac{e^{-ik_b(\rho -\rho^\prime )}}{4\pi |\rho -\rho^\prime |}. \end{aligned}$$

When Eqs. (7b) and (7c) are substituted into Eq. (7a) the resulting equation can be solved for $E^t$. To solve for the superposition found in Eq. (6) we set $J = Sdiag(x)S^T$, which is equivalent to setting all the sources in the superposition on at once with the current density distribution found in *x*. Here *diag*(*x*) creates a matrix of size *M* by *M* with *x* on the main diagonal. This results in

9a $$\begin{aligned} Zx= & {} \sum _{j=1}^{M} z_jx_j = \sum _{j=1}^{M} E_j^t(\rho )x_j = \sum _{j=1}^{M} (E_j^{inc}(\rho ) + E_j^{sc}(\rho ))x_j,\end{aligned}$$

9b $$\begin{aligned} \sum _{j=1}^{M} E_j^{inc}(\rho )x_j= & {} -i\omega \mu _0\left( I + \frac{1}{k_b^2}\nabla \nabla \cdot \right) {\int }_{\rho^\prime  \in bg} G(\rho -\rho^\prime ) Sdiag(x)S^T(\rho^\prime )dV, \end{aligned}$$

9c $$\begin{aligned} \sum _{j=1}^{M} E_j^{sc}(\rho )x_j= & {} \left( I + \frac{1}{k_b^2}\nabla \nabla \cdot \right) {\int }_{\rho^\prime  \in bg} G(\rho -\rho^\prime ) C^{bg}(\rho^\prime ) \sum _{j=1}^{M}E_j^t(\rho^\prime )x_j(\rho^\prime )dV. \end{aligned}$$

Here when Eqs. (9b) and (9c) are substituted into Eq. (9a) we arrive at the remarkable position that we can solve for the unknown *Zx* without explicitly having to calculate *Z*. Now the function that replaces *Zx* for the matrix-free minimization is given by

10 $$\begin{aligned}&\underset{Zx}{min} \left| \left| -i\omega \mu _0\left( I + \frac{1}{k_b^2}\nabla \nabla \cdot \right) {\int }_{\rho^\prime  \in bg} G(\rho -\rho^\prime ) Sdiag(x)S^T(\rho^\prime )dV \right. \right. \\&\quad \left. \left. -Zx + \left( I + \frac{1}{k_b^2}\nabla \nabla \cdot \right) {\int }_{\rho^\prime  \in bg} G(\rho -\rho^\prime ) C^{bg}(\rho^\prime ) Zx(\rho^\prime )dV\right| \right| _2^2. \end{aligned}$$

**Figure 3:**
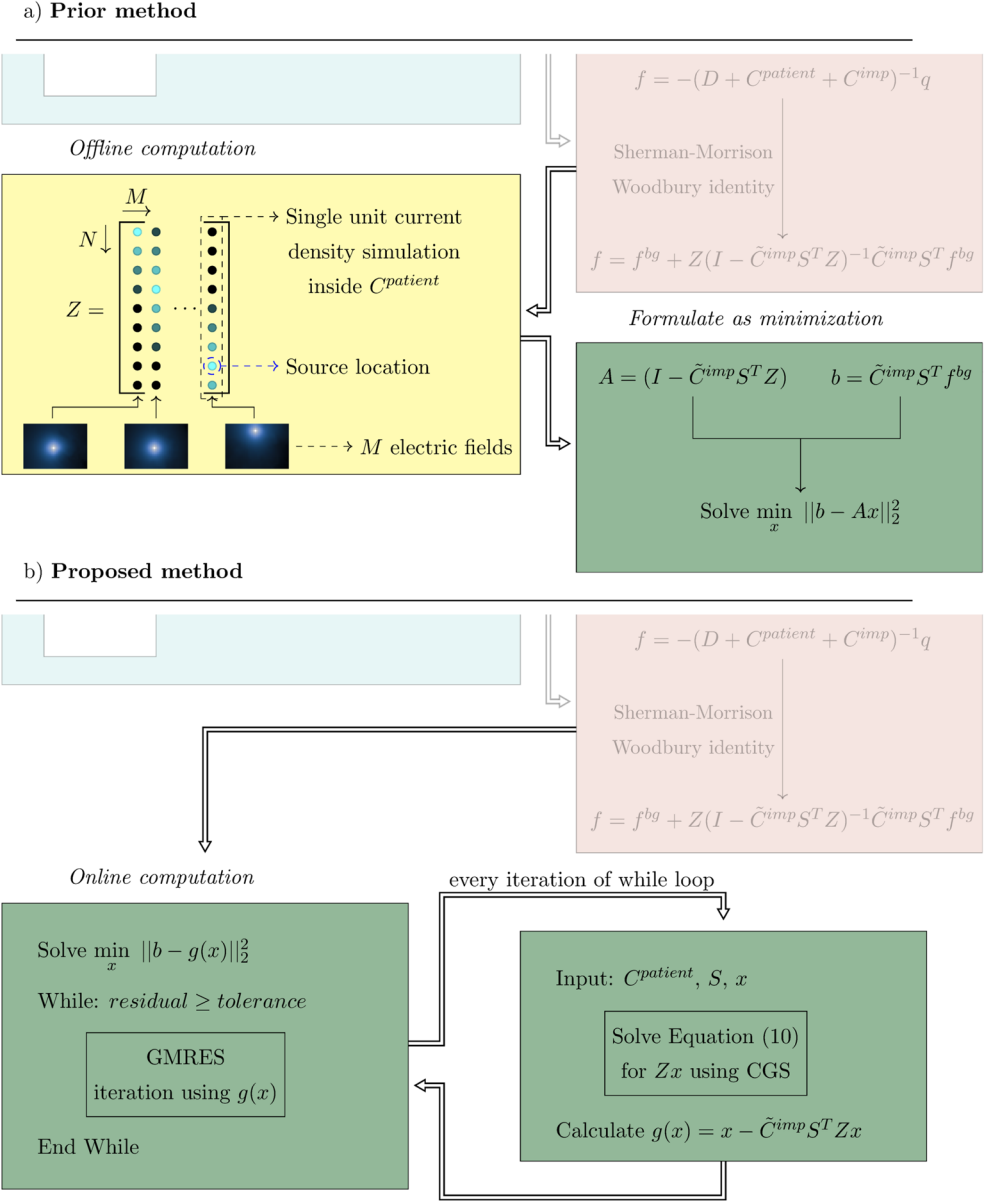
A comparison between the prior and proposed method. (**a**) For the prior method a computationally expensive offline stage is required to obtain the library matrix. Afterwards, the matrix inversion to obtain the RF field distributions resulting from the implant can be solved using a minimization problem. (**b**) We now propose to do the calculation completely online by using a matrix free minimization to compute the matrix inverse. The function that replaces the library matrix is a forward simulation of the RF field inside the patient anatomy generated by sources at the implant location. This forward simulation is performed using the VIE method.

This enables us to solve Eq. (4) without having to compute the massive library matrix *Z*. A schematic visualization of the proposed innovation to the prior method is shown in Fig. 3.

### Simulation results for different medical implants

To verify the correctness of the proposed method, we compare the resulting RF field distributions with those obtained from a traditional FDTD simulation. Four different medical implants are included, where we vary the patient anatomy, implant location, discretization, and the MRI field strength/frequency.

**Figure 4:**
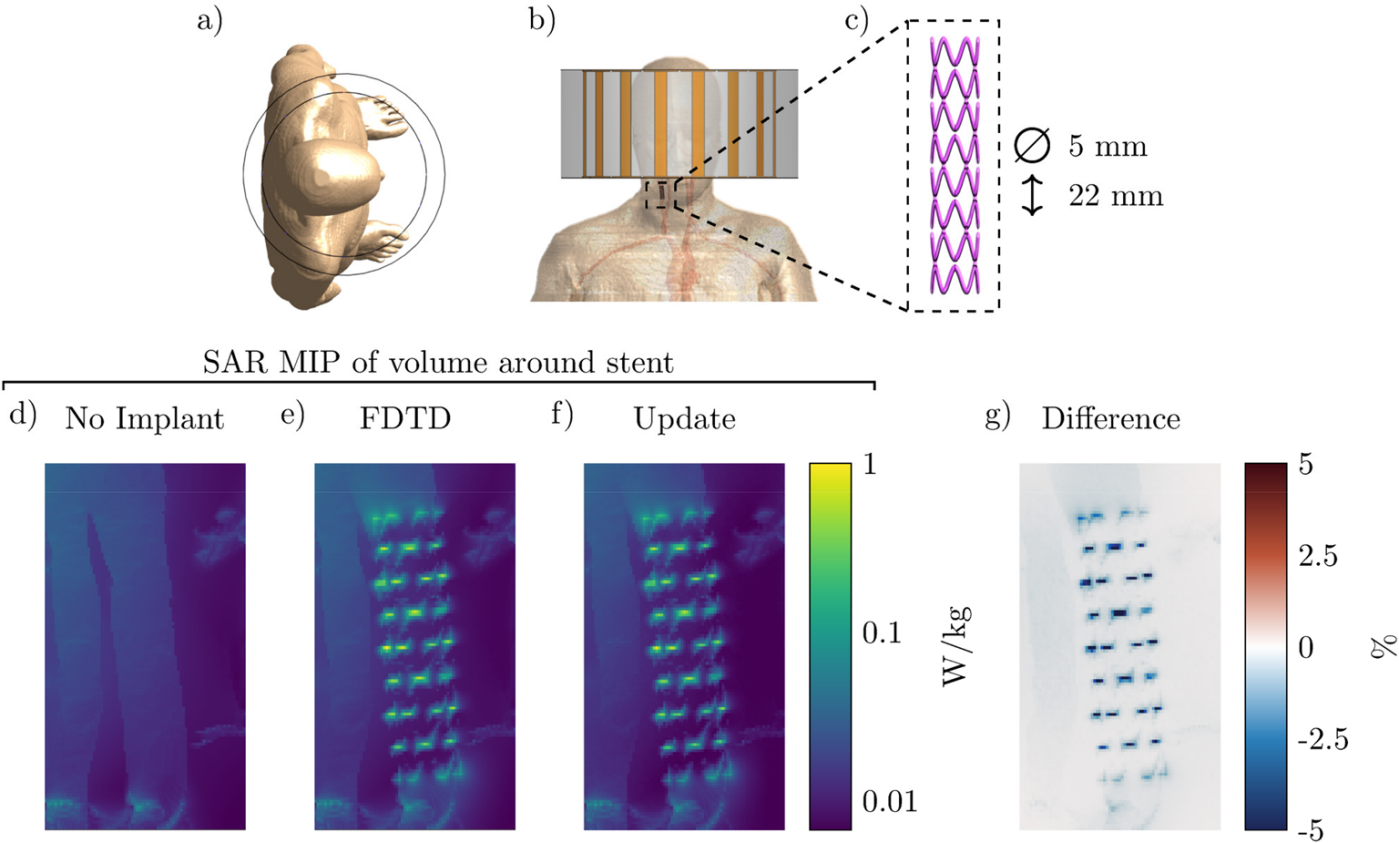
Proposed method applied to calculate the SAR increase for a carotid stent. (**a**) and (**b**) Transverse and coronal view of the simulated setup, respectively. (**c**) The model of the simulated stent. (**d**) Maximum Intensity Projection (MIP) of the local SAR without implant. (**e**) and (**f**) The MIPs of the local SAR with the implant present using the FDTD and the proposed method respectively. (**g**) The MIPs of the difference between the FDTD and the proposed method.

The first implant geometry that we evaluate is a vascular stent placed in the carotid artery where the patient model is placed inside the MRI environment for a head scan at 3 T (128 MHz). The setup and the resulting maximum intensity projections (MIP) of the local SAR distribution can be observed in Fig. 4. Note that the stent causes a 20-fold increase in the peak local SAR. Furthermore, we can observe that the MIP SAR distribution found by the proposed and the FDTD method correlate very well and there is only a small underestimation by the proposed method, as can be seen in the difference distribution in Fig. 4g. The FDTD simulation for this implant took 4 h to run on a GPU while the proposed method took only 96 s (150 times faster). A total of 200 iterations were required to minimize Eq. (4) at 0.475 s per iteration and 1 sec was required for the calculation of the scattered electric field.

**Figure 5:**
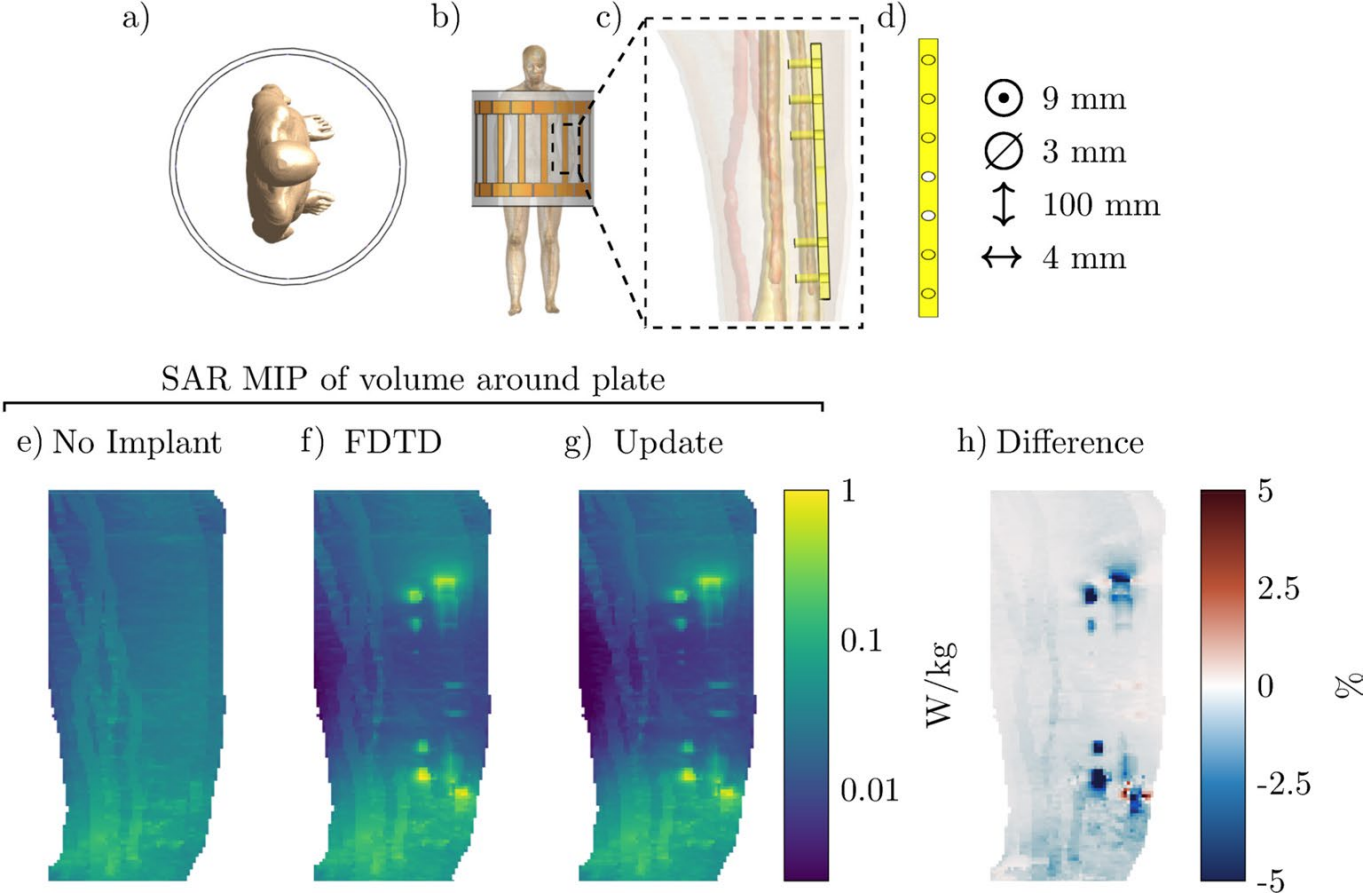
Proposed method applied to calculate the SAR increase for an orthopedic plate with screws. (**a**) and (**b**) Transverse and coronal view of the simulated setup, respectively. (**c**) & (**d**) The model of the simulated orthopedic implant. (**e**) The MIP of the local SAR without implant. (**f**) and (**g**) The MIPs of the local SAR with the implant present using the FDTD and the proposed method respectively. (**h**) The MIPs of the difference between the FDTD and the proposed method.

The next implant for which we test our method is an orthopedic implant placed to hold together the ulna after a fracture. Resulting SAR distributions for a scan at 1.5 T (64 MHz) are shown in Fig. 5. Again, we observe an increase in the peak local SAR values as a result of the implant being present inside the simulation (a 4-fold increase). Furthermore, the MIPs of the SAR distribution from the proposed and the FDTD method correlate well. Here we find both a small under and overestimation of the SAR distribution for the proposed method. For this implant, the FDTD simulation took 3 h and 26 min to converge while it only took 28.2 s using the proposed method (438 times faster). A total of 150 iterations were required to minimize Eq. (4) at 0.185 s per iteration and 0.5 s was required for the calculation of the scattered electric field.

**Figure 6:**
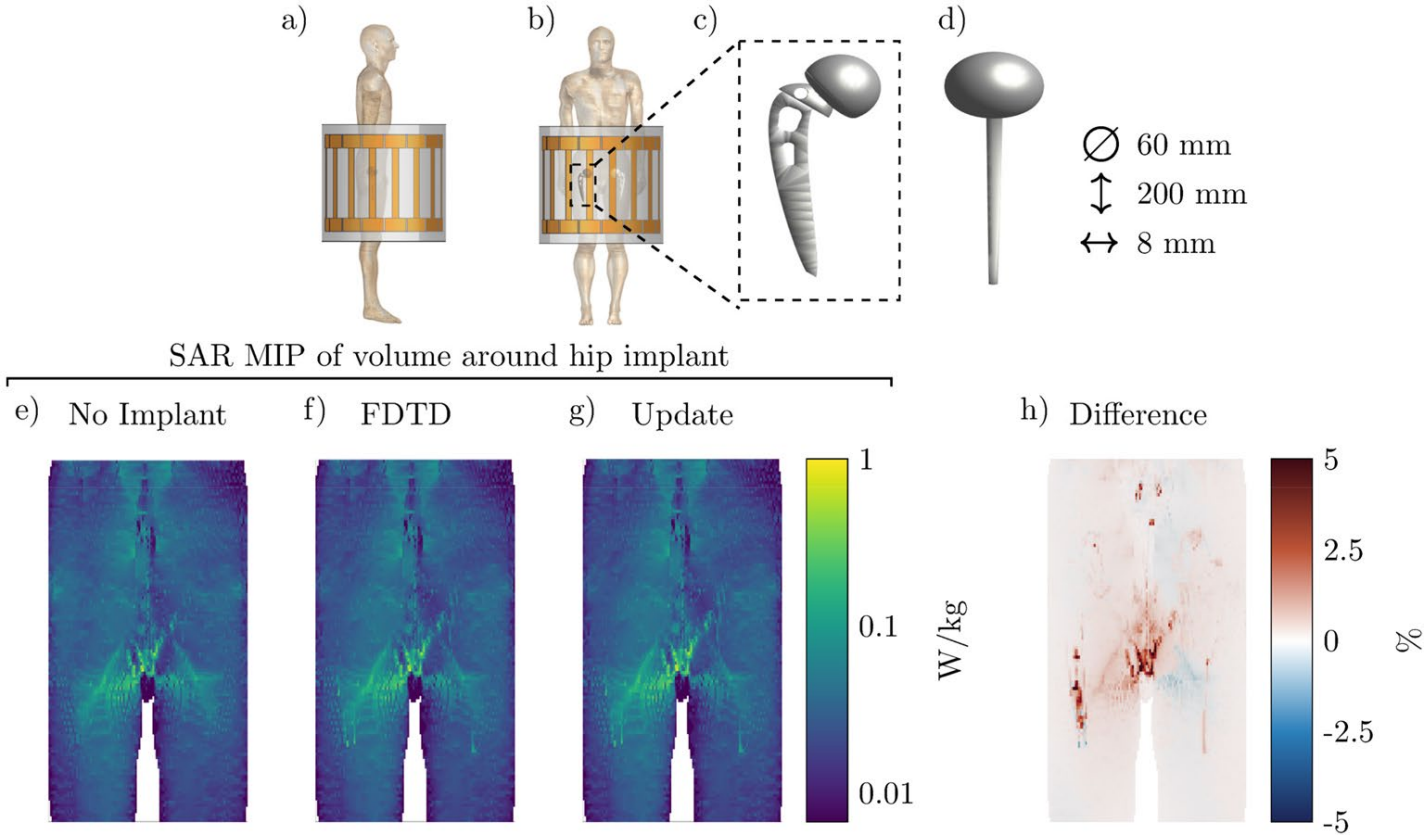
Proposed method applied to calculate the SAR increase for a double hip implant. (**a**) and (**b**) Sagittal and coronal view of the simulated setup, respectively. (**c**) and (**d**) The model of the simulated hip implant. (**e**) The MIP of the local SAR without the hip implant. (**f**) and (**g**) The MIPs of the local SAR with the implant present using the FDTD and the proposed method respectively. (**h**) The MIPs of the difference between the FDTD and the proposed method.

The third implant setup that we simulated is a double hip implant at 1.5 T (64 MHz) for which the setup and MIPs of the SAR distributions are shown in Fig. 6. For this configuration, we observe that there is almost no increase in the SAR values around the implants themselves. Furthermore, the error distribution of the MIP found by the proposed method is smaller compared to the other two simulated setups. The proposed method has converged within 194 s while the FDTD simulation took 3 h, (55 times faster). A total of 250 iterations were required to minimize Eq. (4) at 0.772 s per iteration and 1 sec was required for the calculation of the scattered electric field.

**Figure 7:**
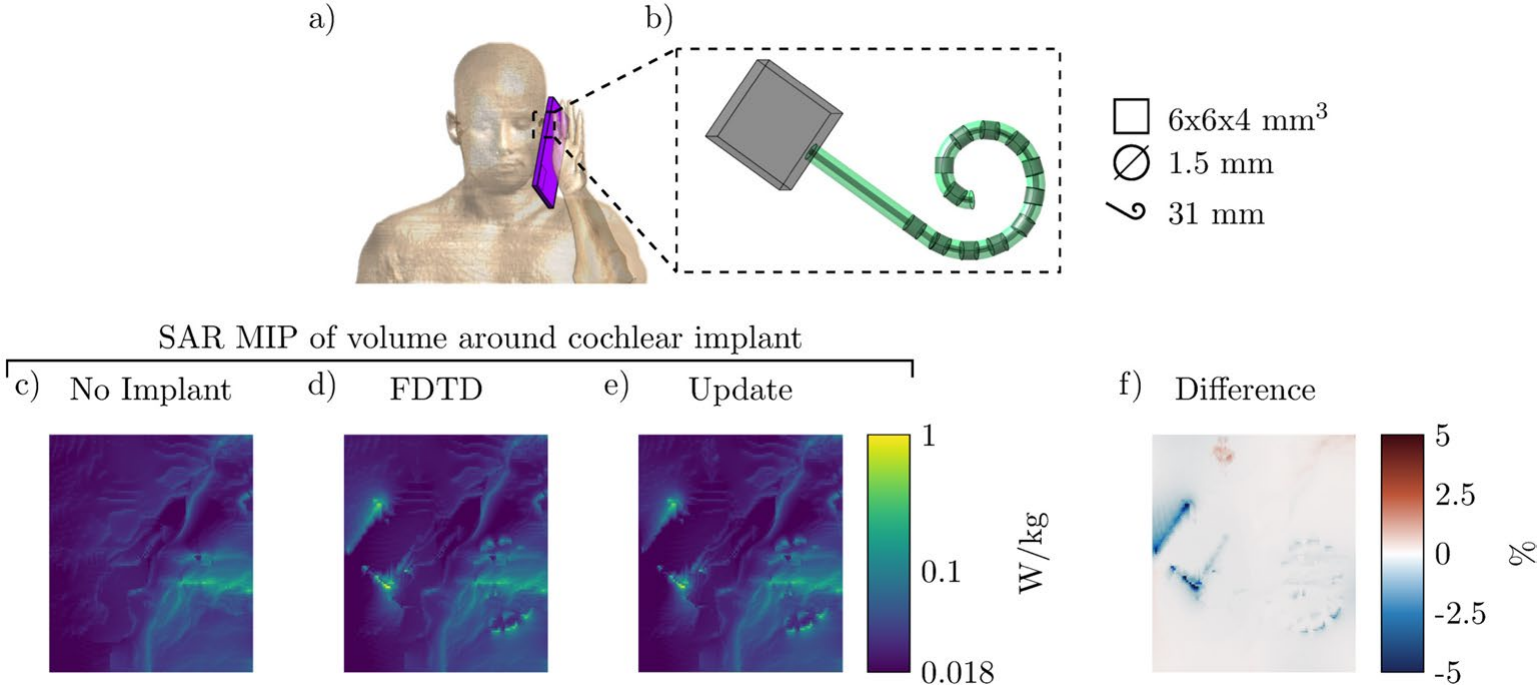
Proposed method applied to calculate the SAR increase for a cochlear implant when using a mobile phone. (**a**) The Ella model with a mobile telephone. The antenna is at the bottom of the phone and operates at 900 MHz. (**b**) The cochlear implant inside the Ella model. The implant has 12 electrodes and the lead is 31 mm long with a diameter of 1.5 mm. (**c**) The MIP of the local SAR without the cochlear implant. (**d**) and (**e**) The MIPs of the local SAR with the implant present using the FDTD and the proposed method respectively. (**f**) The MIPs of the difference between the FDTD and the proposed method.

While the presented method has been developed for the RF safety assessment of medical implants in combination with MRI, it is not limited to this field of research. In fact, Eq. (10) can be solved for any dielectric background, $C^{bg}$ (i.e. not necessarily a patient), or frequency to fit other problem statements. Some examples are computing the change in the power deposition by EM exposure between a standard model and a subject-specific model, the induced SAR by mobile telephones for models with cochlear implants (an example of which is shown in Fig. 7), or any other EM simulation problem where there are sparse changes between simulations. For these other applications, the acceleration factor that is acquired will vary, depending on the size of the update and the domain size reduction. For the cochlear implant, the FDTD simulation took 23 min and 26 s while the proposed method took 66 s (21 times faster). A total of 150 iterations were required to minimize Eq. (4) at 0.436 s per iteration and 0.5 s was required for the calculation of the scattered electric field.

**Figure 8:**
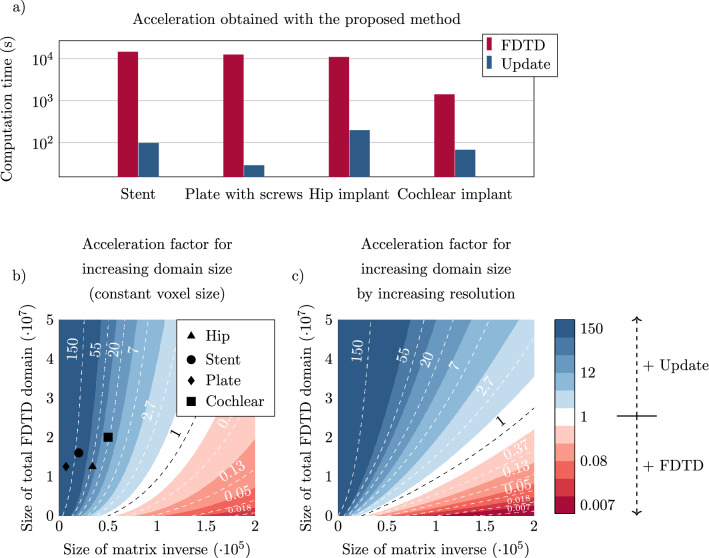
(**a**) Acceleration obtained with the proposed method over the standard FDTD method for the four implants that have been tested. (**b**) Acceleration of the proposed method in comparison to traditional FDTD simulation as a function of the domain size when the voxel size in the FDTD simulation is kept constant. The x-axis of the plot shows the size of the matrix to be inverted (small domain). The y-axis displays the size of the total FDTD simulation domain. To offer perspective on realistic problem sizes, the domain sizes of four implants that are investigated in this work are indicated by markers. (**c**) Acceleration gained with the proposed method as a function of the domain size, however, now the size of the FDTD domain is increased by increasing the resolution. A blue color indicates the proposed method is faster while a red color indicates the FDTD method is faster.

For the investigated cases, the proposed method is orders of magnitude faster, as depicted in Fig. 8. However, it should be stated that, although the proposed method is faster in many scenarios, the improvement will not be realized for particularly large domains. This is because the proposed method and the reference method scale differently with the problem size. The FDTD simulation scales linearly with the number of voxels within the simulation domain and scales inversely with the smallest voxel within the simulation domain. For the proposed method, the computation time required is more complex. The time required to solve Eq. (1) is predominantly used to compute the matrix inversion. The time complexity of this is at worst ${\mathcal {O}}(M^3)$ where *M* is the length of the solution, in our case, this is the number of edges for which the dielectric is changed (i.e. the size of the implant).

As a result, in Fig. 8 an indication of the expected acceleration of the proposed method compared to the FDTD method is shown as a function of the domain size. In Fig. 8(b) the acceleration factor is calculated when the smallest voxel size is kept constant. Figure 8(c) indicates the acceleration when the number of voxels in the total FDTD simulation is increased by increasing the resolution. Because the smallest voxel size is decreased, FDTD characteristics dictate that the simulation time is increased, and the acceleration factor obtained with the proposed method is increased too. Figure 8 shows that at a very large domain size where the implant is present (i.e. *M*/*x*-axis in the plot the FDTD method will be faster than our proposed method.

## Discussion

In this work, we have shown that it is possible to significantly accelerate the calculation of the RF field enhancement that is associated with a medical implant in MRI, without extensive precomputations or excessive storage requirements. This is achieved by using a matrix-free minimization scheme where we use a surrogate function based on the VIE method to replace the intractable so-called library matrix. The local SAR distributions that are obtained using the proposed method are in good agreement with those acquired with a traditionally used FDTD solver.

The acceleration of the proposed method compared to FDTD is obtained by regarding the implant as a perturbation to the original system where the RF field distribution is known. As such, only the effect of the implant on the RF field distributions is calculated.

Using the proposed method, it would be possible to perform patient-specific RF safety assessment for patients with MR conditional implants. The MR conditional label assumes a worst-case scenario which for most MRI examinations would be exceedingly conservative. From the proposed method patient-specific scanning restrictions can be derived to ensure safety while also remaining efficient with the MRI scan time. Furthermore, this method could also be used to assess the RF safety for patients with non-labeled implants. This would allow this patient group to be eligible for MRI examination again, given that the implant is MRI safe on all other aspects. We envision this by using a library of human models and obtaining the implant location from X-ray images acquired prior to the MRI exam and using that information to construct the support matrix *S*. Using this information the SAR near the implant can be calculated for multiple body models and positions of the implant to ensure the SAR increase is not underestimated. Moreover, as a result of the acquired acceleration, we believe that the proposed method is fast enough to enable RF safety assessment online at the MRI scanner. Finally, another application of the proposed method is the so-called Tier 4 RF safety assessment described in the ISO/TS 10794. The accelerated fashion in which the RF field distributions can be calculated will significantly impact the time required for a Tier 4 simulation study.

The acceleration that is found in Fig. 8 also depends on the dimensions of the integrals in Eqs. (9b) and (9c). The fast Fourier transform (FFT) that is performed to efficiently calculate the integrals is computed on a domain size around the implant rather than the entire simulation domain. This FFT domain size will significantly impact the acceleration that is found. The time required for the calculation of the FFTs dominates the time required for the proposed method to converge. Therefore, the proposed method is not efficient when the changes in the dielectric (*M*) are dispersed around the simulation domain (*N*). When the updates inside the domain are dispersed it could be more efficient to apply the proposed method multiple times and apply the update in steps. As an example, the double hip implant update could also be performed in two steps, first the left hip implant and afterward the right hip implant. This would however ignore the interaction that these implants may have on each other.

**Figure 9:**
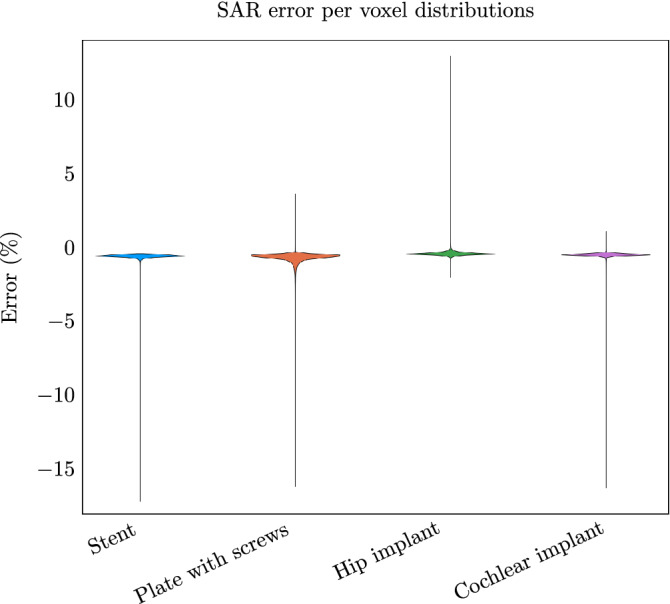
The percentage error distribution for every voxel around the implant. The violin plots show the distribution of the error per implant. A positive error is an overestimation of the proposed method and a negative error is an underestimation.

**Figure 10:**
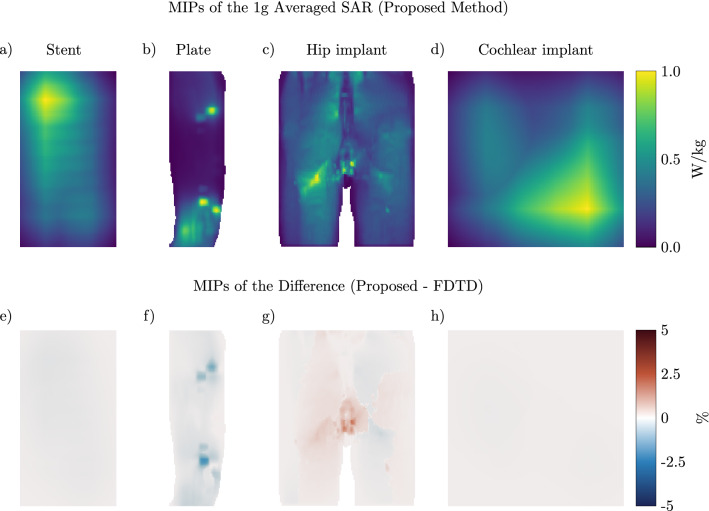
The MIPs of the 1 g averaged SAR obtained with the proposed method (**a** till **d**) and MIPs of the difference between the proposed method and the FDTD simulations (**e** till **h**). (**a**) For the stent. (**b**) For the plate with screws. (**c**) For the hip implant. (**d**) For the cochlear implant. (**e**) The MIP of the difference for the stent. (**f**), (**g**) and (**h**) The same for the plate with screws, hip implant, and the cochlear implant respectively.

From Figs. 4, 5, and 6 it can be observed that the proposed method slightly underestimates the local SAR values. The first reason for the mismatch in the local SAR is that not the entire patient anatomy is considered in the integrals of the proposed method; interaction between the implant and more distant tissue is neglected. For the implants in this manuscript this source of error was minimized by increasing the domain of the FFTs until no decrease of the mismatch between FDTD simulations and the proposed methods were observed. The second reason is that any changes in the current on the RF coil (or $J^{source}$) as a result of a change in loading because of the implant are not included in the proposed method. However, for the investigated implants the RF fields arising from the model perturbation approach zero magnitude at the location of the RF coil. The final reason for a mismatch between the proposed method and the FDTD simulation is that there are inherent differences between the FDTD and VIE methods^34,35^. While the VIE method has been adapted to closely resemble the FDTD method, by having the same Yee cell discretization and dielectric averaging, the method for solving the RF fields is still different and could explain the found underestimation. As for the distribution of the error, only 0.5% of the voxels inside the calculated domain had a larger underestimation than 5% with a maximum of 18% underestimation, this can also be observed in Fig. 9. For our intended application, this is not problematic since the calculated SAR values are on a voxel basis, while all the regulations for MRI implant safety prescribe a 1 g averaged SAR value. Applying such an averaging on the results will decrease the maximum underestimation, as can be observed in Fig. 10. From the MIPs of the differences, it can be observed that the underestimation is decreased significantly compared to the pointwise differences.

In this work, we have reformulated the update method as described in^30,31^ to no longer require the offline stage, and is now completely online. This entails that given an electromagnetic field without an implant present the effect of the implant on the electromagnetic field can be calculated. The proposed method is demonstrated for multiple implants where we show that the calculated RF fields are comparable to the RF fields obtained with FDTD simulations. Using the proposed method we obtain a speed up between 55 and 438 times. Therefore, the proposed method could be used for patient-specific RF safety assessment or an ISO/TS 10974 Tier 4 approach.

## Methods

### Simulation details

The proposed method has been implemented in the Julia programming language^36^ and predominantly uses the existing software packages: CUDA.jl^37^ and IterativeSolvers.jl. The software has been developed into a new package that can be used by others, details are in the code availability section.

To find the solution to Eq. (4) we use a generalized minimal residual (GMRES) method. Multiple solvers have been tested, but GMRES proved to be the most stable and the fastest solver for the problem. For solving Eq. (10) we use the conjugate gradient squared method because it was the fastest solver to obtain the correct solution. Furthermore, the integrals in Eqs. (9b) and (9c) are computed efficiently using 2 FFT operations and a multiplication rather than a convolution which decreases the number of operations from ${\mathcal {O}}(N^2)$ to ${\mathcal {O}}(Nlog(N))$. This can only be performed when the Green’s function is calculated for a homogeneous dielectric background (i.e. a scalar value for $k_b$, vacuum in our case) and the resolution is uniform, note that we do take into account the inhomogeneous dielectric of the patient anatomy using this formulation with the variable $C^{bg}$ in Eq. (9c).

To benchmark our method we use the commercial FDTD package Sim4Life v5.0 (ZMT, Zurich, Switzerland). Furthermore, the body models of Ella and Duke from the virtual family (IT’IS, Zurich, Switzerland) are used.

For each comparison, we simulated a setup without and with the medical implant present. The simulation without the medical implant served as the input RF field distribution for our proposed method, while the simulation with the implant was used to compare the RF field distributions. The medical implants were modeled after standard implant geometries using XCoreModelling within the Sim4Life environment. The metal parts in all implants are modeled as titanium.

In the first comparison, the stent was placed in the carotid artery of the Ella model. The stent is 22 mm long and has a diameter of 5 mm. The thickness of the wires that make up the stent is 0.5 mm. The birdcage head coil that is used has a 155 mm radius, a leg length of 168 mm, a leg width of 20 mm, and the endrings are 2.5 mm wide. The RF shield around the coil has a 190 mm radius and a length of 173 mm. The stent was discretized using a 0.25 mm isotropic resolution and the rest of the setup was automatically discretized by Sim4Life for a total of 15.5 million voxels inside the simulation domain.

For the second comparison, the plate with screws is placed unto the ulna of the Ella model, with the screws going through the bone. The length of the plate is 100 mm long, 4 mm wide, and has a thickness of 2 mm. The screws that are placed inside have a diameter of 3 mm and a length outside of the plate of 9 mm. The birdcage body coil that is used has a radius of 352 mm and a length of 420 mm. The width of the legs is 40 mm while the endrings are 80 mm wide. The RF shield around the coil has a radius of 371.5 mm and a length of 700 mm. The implant is discretized on a 1 by 1 by $2 \,\hbox {mm}^3$ uniform grid and the rest of the setup is discretized using an automatically generated grid for a total of 12 million voxels.

In the third comparison, the two hip implants have a length of 200 mm, a thickness of 8 mm, a width of 30 mm, and the ball has a radius of 30 mm. The two hip implants are identical in size and are placed such that they replace the hip joint inside the body model of Duke. The birdcage body coil in these simulations is identical to the birdcage body coil used for the orthopedic implant placed unto the ulna of Ella. The simulations setup was discretized using 12 million voxels with the hip implants being discretized on a 3 mm isotropic grid.

The last implant that is investigated is a cochlear implant. The lead of the cochlear implant has 12 electrodes, a diameter of 1.5 mm, and is 31 mm long^38,39^. The lead is insulated with a plastic material that has zero conductivity and a relative permittivity of 3. One end of the implanted lead is attached to a casing that is 6 by 6 by $4\,\hbox {mm}^3$. The implant is discretized on a 0.2 mm isotropic grid. In this scenario, the source of the RF fields is a mobile phone antenna. The antenna is a printed inverted F-antenna (PIFA) and operates at 900 MHz.

All the FDTD simulations are terminated at a convergence level of −50 dB and run on an NVIDIA GTX Titan Black. The proposed method uses GPU acceleration for the solver of Eq. (10) running on an NVIDIA RTX 2070 super. The solver for Eq. (4) runs on an Intel Core i5-6600 CPU, but can also run on a GPU. The proposed method is terminated at a convergence level of −50 dB which is similar to the FDTD simulations.

### Theory prior method

For the sake of completeness, here we provide the full details of the original method. A visual overview of the original method can be seen in Fig. 2. We start by discretizing Maxwell’s equations, which describe the generated RF fields during an MRI experiment, given by

11a $$\begin{aligned}&-\nabla \times H + \sigma E + i\omega \varepsilon E = -J^{source}, \end{aligned}$$

11b $$\begin{aligned}&\nabla \times E + i\omega \mu H = 0, \end{aligned}$$

11c $$\begin{aligned}&(D + C^{bg})f^{bg} = -q, \end{aligned}$$

where $\omega$ is the angular frequency, *i* is the imaginary unit, *D* is the discretized version of the curl operators, and *q* contains the source current density ($J^{source}$) which for our application is the current density running through the RF coil. If we now introduce an implant into the problem statement while keeping the discretization the same and solve for *f* our equation becomes

12 $$\begin{aligned} f = -(D + C^{bg} + C^{imp})^{-1}q, \end{aligned}$$

The change that is introduced by $C^{imp}$ takes place only on a small domain compared to the entire simulation domain that includes the RF coil. Using the support matrix *S* we can define the significantly smaller matrix ${\tilde{C}}^{imp}$ as

13 $$\begin{aligned} {\tilde{C}}^{imp} = S^TC^{imp}S. \end{aligned}$$

Equation (12) can now be reformulated using the Sherman–Morrison–Woodbury matrix identity in order to decrease the size of the matrix inverse from *N* by *N* to *M* by *M*.

14 $$\begin{aligned} f&= -(D + C^{bg})^{-1}q \\ &\quad +(D + C^{bg})^{-1}S(I + {\tilde{C}}^{imp}S^T(D + C^{bg})^{-1}S)^{-1}{\tilde{C}}^{imp}S^T(D + C^{bg})^{-1}q.  \end{aligned}$$

When we substitute $f^{bg}$ into the above equation and introduce a so-called library matrix, *Z*, we arrive at

15a $$\begin{aligned} f= & {} f^{bg} +Z(I - {\tilde{C}}^{imp}S^TZ)^{-1}{\tilde{C}}^{imp}S^Tf^{bg}, \end{aligned}$$

15b $$\begin{aligned} Z= & {} -(D + C^{bg})^{-1}S, \end{aligned}$$

where the perturbed RF field distribution *f* is calculated from the unperturbed RF field distributions $f^{bg}$.

When changing the conductivity and permittivity within the simulation domain to include the medical implant, the library matrix only requires the electric field responses of single-source current densities within the patient anatomy. For other applications, using the same formalism and equations as above the magnetic permeability can also be updated in case if the magnetic field responses are required. This could for example be used to calculate the magnetic field distortion around the implant. For this purpose, the magnetic field responses would need to be included within the library matrix after the current density distribution is obtained from the matrix inverse in Eq. (15a). Similar to calculating the electric field using the VIE method once the solution *x* to Eq. (4) is obtained, we can also compute the magnetic field according to

16 $$\begin{aligned} H(\rho ) = H^{bg}(\rho ) + i\omega \nabla \times {\int }_{\rho^\prime  \in bg} G(\rho -\rho^\prime ) Sdiag(x)S^TdV, \end{aligned}$$

where *H* and $H^{bg}$ are the magnetic field distributions when the implant is and is not present respectively, note that since we do not have the library matrix anymore we need an equation to compute the magnetic field part of *f*.

## Data Availability

The code is implemented using the Julia programming language and is supplied as a package for others. This package can be found at: https://github.com/PeterStijnman/DielectricUpdateTechnique.jl.
